# Synergistic motion compensation strategies for positron emission tomography when acquired simultaneously with magnetic resonance imaging

**DOI:** 10.1098/rsta.2020.0207

**Published:** 2021-08-23

**Authors:** Irene Polycarpou, Georgios Soultanidis, Charalampos Tsoumpas

**Affiliations:** ^1^ Department of Health Sciences, European University of Cyprus, Nicosia, Cyprus; ^2^ Biomedical Engineering and Imaging Institute, Icahn School of Medicine at Mount Sinai, New York, NY, USA; ^3^ Biomedical Imaging Science Department, University of Leeds, West Yorkshire, UK; ^4^ Invicro, London, UK

**Keywords:** positron emission tomography and magnetic resonance imaging, motion correction, resolution

## Abstract

Subject motion in positron emission tomography (PET) is a key factor that degrades image resolution and quality, limiting its potential capabilities. Correcting for it is complicated due to the lack of sufficient measured PET data from each position. This poses a significant barrier in calculating the amount of motion occurring during a scan. Motion correction can be implemented at different stages of data processing either during or after image reconstruction, and once applied accurately can substantially improve image quality and information accuracy. With the development of integrated PET-MRI (magnetic resonance imaging) scanners, internal organ motion can be measured concurrently with both PET and MRI. In this review paper, we explore the synergistic use of PET and MRI data to correct for any motion that affects the PET images. Different types of motion that can occur during PET-MRI acquisitions are presented and the associated motion detection, estimation and correction methods are reviewed. Finally, some highlights from recent literature in selected human and animal imaging applications are presented and the importance of motion correction for accurate kinetic modelling in dynamic PET-MRI is emphasized.

This article is part of the theme issue ‘Synergistic tomographic image reconstruction: part 2’.

## 1. Introduction

Scanning a living subject for a reasonable duration affects the quality of the acquired data due to its inevitable motion. Patient motion could be defined as physiological (motion of vital organs i.e. cardiac or respiratory) or spontaneous (i.e. musculoskeletal). This review article addresses the different types of motion that occur during integrated positron emission tomography and magnetic resonance imaging (PET-MRI) acquisitions and considers all aspects relating to the management of motion using potential synergy of PET and MRI data.

### (a) Types of motion

#### (i) Bulk motion of body regions

An area of the body for which motion correction is a common endeavour is the head. In particular, brain PET examinations may last for more than 1 h. The average maximum displacement of the head is 3.9 ± 2.4 mm (1–11 mm) [1] and with respect to the current PET imaging resolution (i.e. 3 mm), motion of this range affects substantially the diagnostic and quantitative accuracy [2–4]. The range of head motion may be even larger in non-anaesthetized children and patients with neurodegenerative diseases, thus emphasizing the need for its correction. More complicated to account for is the movement of the jaw and to a lesser extent the motion of the tongue. Other types of bulk motion involve the arms, shoulders and legs but, compared to the more common PET scanning alongside X-ray computed tomography (PET/CT), this may not be as significant during PET-MRI scanning where the patient is restricted by the associated coils. Finally, the torso itself, although less likely, may exhibit bulk motion [5]. For example, it is well reported that during a PET acquisition the patient may move, due to discomfort, performing a rigid body translation and/or rotation which can also make the internal organs change their position [6], and can be noticeable when scanning patients for substantial duration. Even if in PET-MRI the coils may help reduce these types of motion, claustrophobic patients tend to move during the scan. A past simulation study focusing on the prostate region indicated that lack of correction for bulk motion may create up to 67% error when measuring the standardized uptake value (SUV) [7].

#### (ii) Respiratory

Another common source of motion artefacts is respiration, which creates a displacement and deformation of internal organs (e.g. lungs, liver, stomach, kidneys and heart). As the respiratory motion is not periodic and the path along which the various organs travel during inspiration may differ to the one followed during expiration, the development of a unified approach to correct for it in all acquisitions is not straightforward. The amplitude of diaphragm displacement can be about 15–20 mm during shallow breathing [8]. Motion of this range induces blurring and distortions in images and limits the potential of high-resolution PET-MRI scanners [9,10]. Finally, respiratory motion can affect even organs far from the lungs, such as the brain, by inducing a repetitive motion of the head due to respiration [11]. With the emergence of PET-MRI scanning, there is the potential of synergistic use of MRI to measure respiratory motion more accurately [12].

#### (iii) Cardiac contraction

The heart and the vascular system, beyond respiratory motion, can also substantially be affected by cardiovascular contraction while the ventricles pump blood throughout the circulatory system. Although cardiovascular motion can be more regularly repetitive than respiratory, the contraction mechanism itself makes it particularly difficult to model how each part of the tissue translates during the cardiac cycle. Moreover, patients who experience arrhythmia may have variable cardiac cycles compared to healthy subjects. Finally, modelling this type of motion is more strenuous because it occurs in conjunction with respiration.

#### (iv) Motion of other internal organs

Fluids constantly move within the body. This manifests in a likely expansion of the bladder [13], but also in a change of stomach and intestine locations [14]. Thus, imaging the bladder, the stomach, the intestines and their surroundings can pose additional challenges [15]. For example, in the case of the bladder, its potential expansion and displacement may impact the imaging quality of the uterus, the ovaries and the prostate. For relatively short imaging acquisitions, this type of motion may not be so substantial when compared to PET resolution, but it would be more profound in longer acquisitions. For example, a typical multiple bed-positions static scan of a patient in a PET scanner lasts around twenty minutes. However, if the scan is dynamic, the acquisition will most likely focus on one bed position which could last longer than an hour. To the best of our knowledge, there have been no PET-MRI investigations that have attempted to correct for such physiological expansion of the bladder or other internal organs, yet.

### (b) Motion management

**Figure 1.**
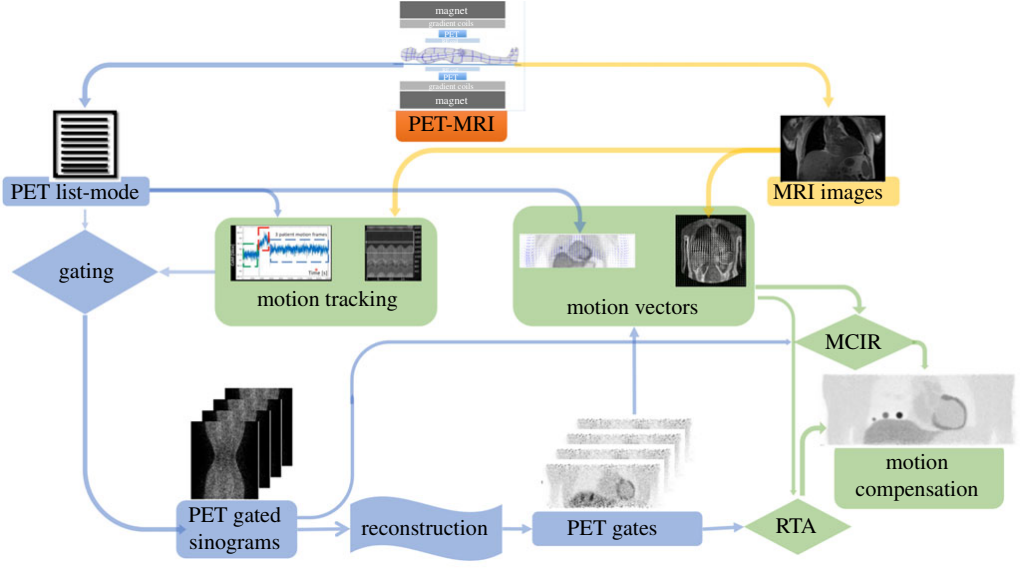
Diagram of motion management strategies in PET-MRI. Motion can be tracked by creating surrogate signals from PET and/or MRI. Likewise, motion vectors can be created from both modalities. PET data can be subdivided in short frames/gates which include only a small fraction of motion. Motion correction of the PET data can be achieved by integrating motion vectors within reconstruction (MCIR) or by applying them to previously reconstructed images (RTA). (Online version in colour.)

Motion can affect PET-MRI imaging in many different ways. It reduces resolution and creates artefacts which pose a burden for accurate diagnosis [9,16]. Furthermore, it degrades quantification, which is an important issue when used for disease grading and may directly impact treatment planning and response assessment. All these limitations can be controlled by addressing each type of motion properly and many strategies have been implemented for this purpose as shown in figure 1. Moreover, it is worth highlighting a somewhat counterintuitive fact: motion can improve the imaging system resolution because it increases spatial sampling. For example, a tiny rotation (equal to half the size of the crystal) of PET scanners was commonly applied in the past for increasing the spatial sampling and consequently the resolution [17]. Therefore, proper motion management can help increase the resolution of the imaging system due to improved sampling, compared to motion-less acquisitions, an effect also referred to as super-resolution [18].

A traditional method to achieve image capture without motion artefacts is asking the subject not to move, e.g. hold their breath. This is common practice in X-ray or CT scanning and less common in MRI [19]. In PET imaging, currently this is not the case, though it may well become in the future if scanner sensitivity increases substantially. For example, it has been shown that PET images of reasonable quality can be captured within 30 s per bed position with a recent time of flight PET/CT scanner [20]. However, in the near future, we do not anticipate this to be the way of achieving motion correction in PET-MRI mainly because MRI acquisitions are longer than CT, and such gain in PET sensitivity is more likely to be used as a means to reduce the injected dosage into the patient or improve the signal-to-noise ratio (SNR). Nevertheless, it is not unlikely that a breath-hold acquisition protocol may come to accompany a longer free-breathing protocol where the two acquisitions may be used to produce a high quality motion corrected image.

A common method to minimize repetitive types of motion (e.g. respiratory or cardiac) which can be approximated as periodic is the ‘gated’ acquisition. Respiratory gating uses a surrogate signal based on respiratory amplitude or phase and separates the data into multiple gates. The surrogate signal can be derived by various MRI sequences, including one-dimensional and two-dimensional navigator acquisitions [21]. Cardiac gating is separated based on cardiac phases usually provided by an electrocardiogram (ECG). Gating itself does not correct for motion but simply accepts data within a limited time window set by the surrogate signal. In this way, it excludes substantial motion in each gate with the assumption that there is only small motion during the entire time window of the surrogate signal and that the not-so-periodic pattern can be assigned accurately to the corresponding gate. Sorting data into gates has an additional caveat: the gated images suffer from substantially lower SNR than when using all data, which strongly depends on the motion pattern and consequently the duration of each gate. This restriction is even more evident when imaging the heart or its surroundings, which are affected by multiple types of motion. Due to this important limitation of gating, a technique correcting for motion which uses all measured data in order to recover the SNR may provide superior results. One approach for motion correction of gated data consists of two steps: estimation of motion and then correction of each gated image separately [22].

## 2. Motion tracking

One of the first steps involved in correcting for motion is ‘motion tracking’. This step can be used for rebinning the data into motionless frames and/or gates from which, once reconstructed, the motion vectors can be calculated using image registration algorithms [23]. Additionally, motion tracking can be used as a surrogate signal to create a motion model and it can be obtained either from MRI, or from PET or more recently from both PET and MRI [24]. Alternatively, external devices can be used to track motion such as optical probes, particularly for brain imaging [25], or pressure bellows for imaging the thorax or abdominal regions [26].

A certain MRI sequence (i.e. tagged MRI) has been applied as a motion tracking technique in PET-MRI [27]. During tagged MRI, temporary features (i.e. tags) are created on the muscle of interest and its deformations due to motion become visible. However, while MRI tagging has been successfully demonstrated in animal imaging [27], it may not be so practical for imaging certain types of organs in humans due to the potential fading of the tags. For example, in the area of the liver, T1 is approximately 0.8 s at 3T which is substantially shorter than the duration of a human typical respiratory cycle (4–7 s) and therefore the tags will fade too quickly to capture any respiratory motion.

The gold standard of tracking and measuring respiratory motion is considered to be the MRI navigator [28]. The appropriate use of navigator echoes helps track the displacement of the diaphragm. The common applications of navigators can be one-dimensional, known also as pencil-beam navigator, two-dimensional or three-dimensional navigators. A common one-dimensional navigator can be extracted from the right hemidiaphragm at the lung-liver interface. While a patient breathes freely, the navigator-echoes track the craniocaudial motion of the diaphragm over time without any substantial interference from other acquired sequences. The expected spatial resolution can be as high as 0.5 mm with a 10 ms temporal resolution. The limitation of a pencil-beam navigator lies in the constrained field of sampling [29]. Such pencil-beam navigators may be encapsulated in a golden angle radial FLASH pulse sequence to obtain time-dependent MRI images as applied in pulmonary PET-MRI [30]. Two-dimensional navigators are in use to correct for respiratory as well as head motion [31]. The spatial resolution of a two-dimensional navigator can be at 1.8 × 1.8 mm^2^ with an expected temporal resolution no better than around 185 ms [32]. One advantage of the two-dimensional navigators is that multiple one-dimensional navigators can be extracted from distant points providing multiple surrogate signals for motion tracking, or an input for a more complete fully three-dimensional respiratory motion model [33]. The disadvantage of a two-dimensional navigator lies in the out-of-plane motion which a three-dimensional navigator can resolve effectively. By capturing the entire volume with a three-dimensional navigator, however, the temporal and spatial resolution is sacrificed which can be translated into an expected voxel size of only 7.5 mm isotropic and temporal resolution at 300 ms. The different methodologies of one-dimensional and two-dimensional navigators for the comparison of techniques of motion tracking and their expected temporal resolution have been presented in the first table of the review article by Paganelli *et al.* [34].

An application of respiratory motion tracking via navigators is a sequence known as ‘NAV-TrueFISP’ which can measure the respiratory motion of the lower abdomen [35]. This takes advantage of single-slice steady-state free precession MRI acquisitions (TrueFISP) interleaved by pencil-beam navigator echoes. The navigators are collected prior to each acquired slice with TrueFISP followed by tracking of the lung-liver interface in that corresponding slice, thus monitoring the respiratory cycle. The obtained motion surrogates can be used for rebinning the PET-MRI data into respiratory phases while the MRI gated data can be used to compute the motion vectors. However, this approach is currently only advised when there are no substantial artefacts from other types of motion. In order to track motion the surrogate signal is located in certain positions (e.g. interface between the lung and liver to measure the respiratory motion) and if the motion is outside the region of the surrogate it cannot be captured.

Another method used the MRI component of PET-MRI to produce respiratory surrogate signals by having an external device to emit a specific radio frequency. This is known as the ‘pilot tone’ where a single-frequency signal, emitted by a portable device, is modulated within the human body. The presence of respiratory motion creates a temporal variation of this modulation, and it can be used for tracking. The benefit of this technique is that the used frequency is outside the MRI readout band but within the RF receiver capabilities and therefore can be executed in the background, along with standard MR sequences [36].

The use of MRI for motion tracking is not necessarily the rule. In recent studies, Kesner *et al.* demonstrated the capability to perform respiratory motion tracking by using PET list-mode data. This aspect allows MRI to perform other significant tasks [37]. Likewise, Lassen *et al.* tracked motion via PET list-mode in coronary PET images [38]. With this methodology, the authors can track any potential bulk motion of the patient during the scan.

Finally, a synergistic approach has recently been implemented by Mayer *et al.* where they used mutual information from PET and MRI to both derive the motion vectors and correct for motion [39].

## 3. Motion estimation

The potential of using MRI for clinical imaging as well as motion tracking has designated motion correction for PET-MRI a challenging and exciting research area. Scientists are trying to develop sequences that can operate in an interleaving fashion with other sequences or motion models which can help predict motion at any given time during the acquisition. An example of parallel sequences is given by Johnson *et al*., who incorporated six degrees of freedom motion tracking spherical three-dimensional navigators into a turbo-FLASH sequence with no detrimental impact to image quality [40]. These models use minimal MRI information to correlate each motion of different parts of the body with a surrogate signal, which can be obtained from an external device or another MRI sequence—and this is why we refer to them as hybrid tracking methods. For example, King *et al.* developed a motion model from real-time MRI acquisitions driven by dynamic signals [21]. Similarly, a joint PET-MRI motion model was introduced by Manber *et al.* [41] to correct for motion [42].

Estimation of motion is usually achieved by appropriate registration of images with a selected reference image. Image registration can be applied to series of dynamic images obtained from appropriate acquisitions such as tagged-MRI [27,43] or two-dimensional multi-slice MRI techniques [44–46]. Tagging sequences, apart from the rapid fading of the tags as mentioned previously, are disadvantaged by prolonged acquisition and this depends on the required resolution. For example, Huang *et al.* used tagged MRI and for the specific resolution parameters, the scan lasted more than 8 min [47]. On the other hand, the temporal resolution of two-dimensional multi-slice gradient echo techniques may depend on the location of the acquisition, e.g. in the torso its temporal resolution varies between 400–700 ms [44,46,48]. An alternative method proposed to acquire fast two-dimensional axial images in random respiratory positions sorted retrospectively to the corresponding respiratory phases depending on their amplitude [49]. The sequence selection is based upon the short acquisition time and optimization of image quality.

To handle the distorting effects of motion simultaneous imaging of four-dimensional PET-MRI is required over several respiratory and cardiac cycles which then need to be sorted into gates. The principal disadvantage of this method is that MRI would be used solely as a motion correction technique for PET, thus failing to provide valuable clinical information. Moreover, MRI is not necessarily fast enough to acquire three-dimensional images of sufficient resolution, SNR and contrast within 100–200 ms frame duration. Therefore, this approach has not been considered apart from a feasibility study demonstrating the potential of correcting the PET images for motion with information obtained from an integrated PET-MRI prototype [50].

The reconstruction of high-resolution MRI volumes at a high frame rate may help handle situations with even more complex motion such as arrhythmia, irregular breathing or bulk non rigid motion to the potential expense of SNR. Appropriate MRI sampling schemes, for example the radial phase encoding, can be used to reconstruct images at different temporal resolutions from the same acquired data and therefore create images with high temporal resolution [7]. More recently, real-time MRI acquisitions were proposed to estimate motion using subspaced-based MRI from highly under-sampled k-space data [5]. A method to estimate motion with potentially shorter temporal resolution than its acquisition may be possible by motion modelling and real-time transformation calculations [51]. The first step requires the acquisition of a dataset during the respiratory cycle with high spatial and sufficient temporal resolution. This dataset could be used to train a motion model algorithm. Subsequently, pencil beam navigators, single two-dimensional slices or multiple two-dimensional slices can be acquired with high temporal resolution. The image features are correlated with the motion model in order to calculate a three-dimensional motion vector representation. Therefore, the appropriate use of motion models can computationally improve the temporal resolution of MRI and consequently PET [21,52].

Image registration can be applied globally to the entire image or locally to a specific region. The choice of appropriate image registration algorithms depends on the type of deformation. These algorithms can be categorized in the type of motion they deal with such as rigid, affine or free-form. The efficiency and accuracy of image registration algorithms play a key role in the end result of motion correction, thus any method needs careful validation prior to its use. Grand challenges such as the EMPIRE10 (https://empire10.grand-challenge.org/) provide helpful means to cross-compare the overall performance of the algorithms in certain conditions.

## 4. Motion correction of the attenuation map

Beyond the blurring of the reconstructed images, respiratory motion can also cause spatial mismatch with the independently acquired and/or synthesized attenuation map, which is usually derived from MRI acquisitions [53]. In PET-MRI, the attenuation map is also expected to be affected by motion because the corresponding sequences used as input information to derive such maps can take a considerable duration to acquire. Therefore, a rigorous PET motion correction regime ought to correct for any motion which affects the attenuation image. In one recent investigation, gated MRI attenuation maps were generated by acquiring data under free breathing, sorting them according to their respiratory phase and using them afterwards to correct for the attenuation of the respiratory gated PET data [54]. An alternative approach could focus on the estimation of motion vectors from dynamic non-attenuation corrected PET data and apply them in order to warp an attenuation map of a single respiratory position to various respiratory gates [55]. Motion-induced artefacts of the attenuation map are not straightforward to resolve as the attenuation values can depend on the size of the deformation. For example, lung density variation during respiration can affect the attenuation values of the corresponding region substantially [56–58]. Holman *et al.* quantified the effect of density mismatch in the area of lungs when applying CT attenuation correction in PET images and found errors of up to 25% in the estimation of SUVs [56]. It is helpful to note that the calculation of the attenuation map can affect scatter estimation, as for example can happen when there is substantial motion of the arms or legs if these are not tight with the coils [59]. Finally, the use of coils can create attenuation artefacts if they move during the scan, which is not uncommon particularly if they are flexible, thus calculating the transformed attenuation map of the corresponding coils would involve an additional step in the whole process [60]. Consequently, attenuation mismatches due to motion can disrupt the reconstructed image in various ways and correcting for the motion of the attenuation map is an essential element for producing motion compensated PET-MRI images.

## 5. Motion correction methodologies

Motion information is mainly used in two different approaches for motion correction of PET data: during or after image reconstruction known also as ‘Reconstruct Transform Average (RTA)’ and ‘Motion Compensated Image Reconstruction (MCIR)’, respectively. In RTA, each gate is reconstructed independently and transformed into one reference gate and then all gates are averaged accordingly. On the other hand, in MCIR, the image transformations are incorporated directly into the reconstruction algorithm and data from all gates are processed altogether to create a motion corrected image.

### (a) Post-reconstruction methods

RTA is a simple approach that manufacturers have already incorporated in their software and it has been applied to PET-MRI data for cardiac [61], respiratory [44] and cardio-respiratory motion models [62]. The main disadvantage of this technique is that in the presence of insufficient measured data in any gate, there will be inherent bias due to the non-negativity constraints of the mainly used reconstruction algorithm as shown by Polycarpou *et al.* [63]. This bias exists in all low count gates and will be accumulated to the averaged motion corrected image. Another disadvantage of the RTA is that the transformation of the reconstructed images further degrades the resolution of the final image due to the application of consequent image-based interpolations [63]. Nevertheless, in most cases, RTA remains a practical approach because there is no need to modify the available commercial software.

### (b) Methods within image reconstruction

On the other hand, MCIR incorporates the interpolators in each iteration and the resolution is not affected. In PET-MRI, MCIR has been applied in order to resolve head [64], heart [35] and respiratory [43,65,66] motion. Many other studies compensated for respiratory motion using time/gate-varying system matrix in the reconstruction [27,43]. Dikaios *et al* compensated for respiratory motion by incorporating motion information prior to forward-projection and following backward-projection steps of the reconstruction algorithm [67,68]. All these studies performed motion correction using gated data. Alternatively, motion information can be incorporated as part of a more complete motion model. Using MRI data Kustner *et al.* [69] created a motion model and attenuation map that were fed to a reconstruction algorithm. Manber *et al.* [65] extracted the respiratory signal directly from PET and used it to gate the data and construct a motion model in combination with dynamic MRI. The estimated motion was incorporated into PET reconstruction to obtain a single motion-corrected image. MCIR was also demonstrated with motion estimated from undersampled MRI acquisitions as fast as 1 min per bed position [66]. In particular, undersampled MRI acquisitions for estimating motion can accelerate the acquisitions making them desirable in clinical practice as they can allow the acquisition of additional sequences within the same scanning session [70].

## 6. Applications of motion correction

Motion correction enhances early detection and accurate staging of disease for more successful and cost-efficient treatments [9]. In the following paragraphs, we illustrate PET-MRI application areas that can clearly benefit from motion correction.

### (a) Brain imaging

**Figure 2.**
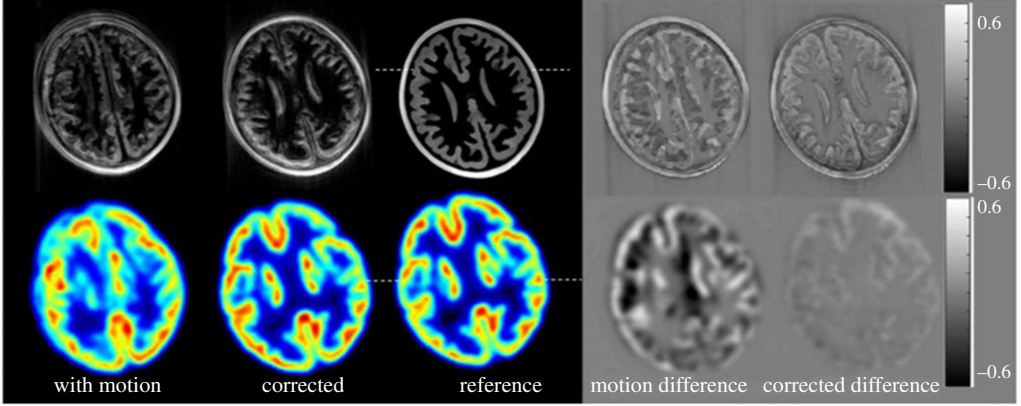
Motion correction results. A single slice of the motion-corrupted, corrected and reference PET and MRI images are shown. The difference images are presented in the fourth and fifth columns. The colour bars indicate the range of pixel intensities in the difference images (original images were scaled from 0 to 1). (Reproduced from Johnson *et al.* 2019. Rigid-body motion correction in hybrid PET/MRI using spherical navigator echoes. *Physics in Medicine and Biology*, **64**, doi:10.1088/1361-6560/ab10b2 © Institute of Physics & Engineering in Medicine. Reproduced by permission of IOP Publishing. All rights reserved). [40]. (Online version in colour.)

Motion correction has been investigated widely by various brain PET-MRI studies [1,3,71–73]. An example of motion correction for brain PET-MRI is illustrated in figure 2. Although not fully used, it is anticipated that brain PET-MRI will be the first application area where motion correction will be routinely incorporated because it has been already commonly used in MRI-alone clinical systems. The main difference in brain PET versus the typical body applications is the duration of the study, which can last for much longer, and a need for absolute quantification especially if kinetic analysis is envisaged. Therefore, precise motion estimation and compensation throughout the study is essential and the need for MRI is important especially at the early stages of the acquisition where PET has a very limited signal. Furthermore, studies may require kinetic modelling to extract physiologically more meaningful parameters than SUV. Such studies may need to calculate the arterial input function from images. This information could be extracted from regions such as the carotid arteries, and motion correction is even more important to obtain reasonable estimates from these small vessels. Furthermore, dedicated brain PET-MRI scanners are capable of acquiring images with higher spatial resolutions [74] than commercial clinical PET-MRI scanners (e.g. 2 mm versus 4 mm), thus motion compensation becomes one of the key limiting factors for looking deeper in small brain sub-regions. High-resolution brain PET-MRI is a fast-evolving area with many recently sponsored research projects in the world and it is expected to deliver more concrete motion compensation approaches in the coming years.

### (b) Cancer imaging

Motion correction is essential for detecting small and/or low uptake tumours [9] in many different regions of the body, and consequently contributes to more accurate image interpretation and clinical decision making [35,42,65,75]. The impact of motion correction in particular has been investigated for thorax or pulmonary imaging which are areas greatly affected by respiratory motion. After motion correction, contrast is increased by up to 50% for lung lesions [30] and 45% for hepatic lesions [35]. In addition, studies have reported a possible reduction in the size of the lesions [35,76]. Petibon *et al.* investigated patients with hepatic lesions showing a reduction in size ranging from 12% to 29% [35]. Another study reported a reduction of the volume of liver lesions up to 29% after applying motion correction [77]. Studies have also shown that motion correction achieved a more accurate lesion delineation [35,69] that is expected to enhance diagnostic accuracy.

**Figure 3.**
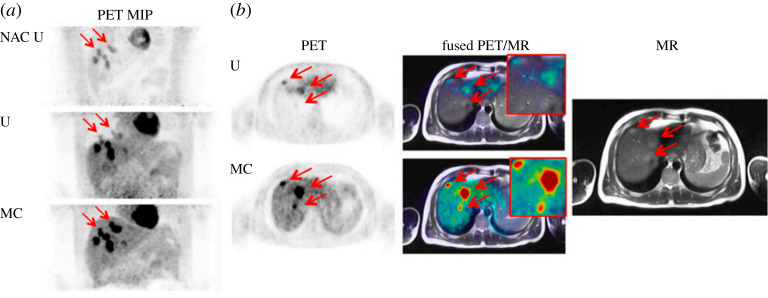
[^18^F]FDG PET-MRI study. (*a*) Maximum-intensity projection (MIP) non–attenuation-corrected, non-motion-corrected (NAC U); attenuation-corrected, non-motion-corrected (U); attenuation-corrected & motion-corrected (MC) PET images. (*b*) Axial PET slices with three lesions (arrows) that wrongly appear in the lungs in the uncorrected image (U) and correctly appear in the liver in the motion-corrected image (MC), along with fused T2-weighted half-Fourier–acquired single-shot turbo spin-echo MRI-PET images and MRI image alone. (This research was originally published in JNM. Manber, Thielemans, *et al.* Clinical impact of respiratory motion correction in simultaneous PET-MRI using a joint PET/MRI predictive motion model. *The Journal of Nuclear Medicine* 2018; **59**, 1467–1473. © SNMMI) [42]. (Online version in colour)

**Figure 4.**
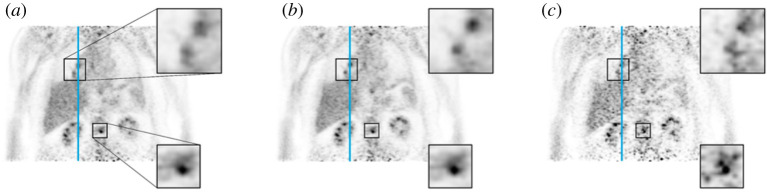
Visual comparison of PET images as obtained by: uncorrected data (*a*), motion correction (*b*), and gated reconstruction (*c*) containing a (motion-affected) lesion next to the hilum and a (static) osseous lesion in the lower spine. Magnified regions around these lesions are shown in the adjacent images. Note the enhanced sharpness and signal-to-noise ratio of the hilar lesion in the motion corrected data in comparison to the other two methods, while the bone lesion in the region with less motion varies less between the uncorrected and motion compensated reconstructions (Reproduced from Gratz M, Ruhlmann V, Umutlu L, Fenchel M, Hong I, Quick HH (2020) Impact of respiratory motion correction on lesion visibility and quantification in thoracic PET/MRI imaging. *PLoS ONE* **15**, e0233209) [78]. (Online version in colour.)

Any possible improvement after motion compensation depends on the degree of motion and the area investigated. In particular, lesions that are located in boundaries of organs may be misplaced due to motion. Manber *et al.* in PET-MRI [^18^F]FDG studies demonstrated that lesions wrongly appeared in the lungs prior to motion correction, while they appeared correctly in the liver following motion compensation (figure 3) [42]. Another study reported that after motion correction, an additional lesion was identified in the liver [77]. More specifically in a recent investigation concentrated on patients with lung lesions, the readers scored lesions in the motion corrected images with 38% and 56% more confidence compared to the non-corrected images and gated images, respectively (figure 4) [78].

Despite its recognized impact in oncology, motion correction is primarily used for research purposes as its routine use is hampered by various issues (e.g. computational resource & time demand). While many PET-MRI studies focus on lung and liver lesions, other areas can benefit from motion compensation in oncology. For instance, in a study for the assessment of metastatic lesions in PET-MRI of the head and neck, data were corrected for any possible motion (i.e. swallowing or breathing) prior to any further analysis [79].

In oncology, although static PET acquisitions are conventionally used, in some clinical scenarios dynamic acquisitions could provide clinically useful information. In PET-MRI scanners, the kinetic analysis can be further enhanced compared to PET/CT due to the complementary simultaneous MRI acquisition which allows the extraction of motion parameters that can be used to correct the PET data for motion prior to the calculation of any kinetic metrics.

### (c) Cardiovascular imaging

**Figure 5.**
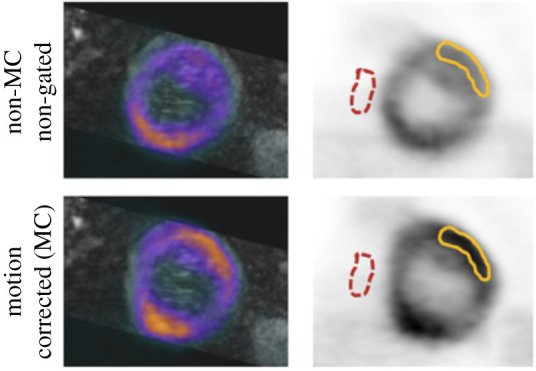
Identifying regions affected by motion: blurring and signal loss within the myocardium when non-motion corrected (non-MC) non-gated compared to motion corrected (MC) PET images as in the antero-lateral wall (solid line). Background regions were drawn in the blood pool on the right or left ventricle (dashed line). (Reproduced from Robson PM, Trivieri MG, Karakatsanis NA, Padilla M, Abgral R, Dweck MR, Kovacic JC, Fayad ZA. (2018) Correction of respiratory and cardiac motion in cardiac PET/MRI using MR-based motion modelling. *Physics in Medicine and Biology* **63**, doi:10.1088/1361-6560/aaea97 © Institute of Physics & Engineering in Medicine. Reproduced by permission of IOP Publishing. All rights reserved). [62]. (Online version in colour.)

Motion correction in cardiovascular imaging has been proven to be beneficial. In PET-MRI cardiac studies correcting for cardiac contraction only was able to provide an increase of up to 50% in the SNR compared to the non-corrected images [80]. Robson *et al.* used RTA to resolve motion in PET-MRI data of patients with cardiac sarcoidosis. Following correction for both respiratory and cardiac motion the contrast of the images was clearly improved as illustrated in figure 5 [62]. Motion correction for more accurate evaluation of the myocardial viability has been performed by Munoz *et al.*, who tracked respiratory motion as well as calculating the motion vectors from MRI and applied them to PET images using the RTA method for respiratory [81] and cardio/respiratory motion [82]. Another challenging area to apply motion correction is in the coronary arteries as in the paradigm illustrated via a computational simulation study by Petibon *et al.* [83]. The advantages of the synergistic use of PET-MRI for respiratory and cardiac motion correction have been visible in recent clinical studies where it was shown that the visualization of uptake in coronary plaques can be improved [39].

Finally, it would be remiss not to state that similar to brain imaging, kinetic modelling can be essential in extracting valuable physiological information on the cardiac function. However, it is implicit that without a robust and well-tested methodology for motion compensation of cardiac imaging for all different types of motion (i.e. respiratory, cardiac contraction and bulk movements), sophisticated kinetic models are of limited value especially if focused on small regions. Improvements in kinetic quantification have been reported by Petibon *et al.* for dynamic cardiac PET-MRI after correction for cardiac and respiratory motion prior to kinetic analysis [84]. More than that, imaging the blood pool or the arterial input function from the ascending and descending aortas can be used as image-deriv ed input function in kinetic modelling of any regions in the corresponding field of view. This creates another important potential advantage by achieving an accurate, though challenging, motion compensation of the cardiac cavities and the adjacent aortas.

Finally, Kesner *et al.* and Vahle *et al.* demonstrated that respiratory motion tracking is feasible without an additional burden to the MRI scanner [36,37]. Recently, accelerated MRI-based motion field measurements [85] and the use of deep learning towards MRI motion correction [86] offer potential alternative directions for motion estimation. All these tools can be used in conjunction with each other towards the improvement of quality in motion tracking and estimation.

## 7. Animal imaging

**Figure 6.**
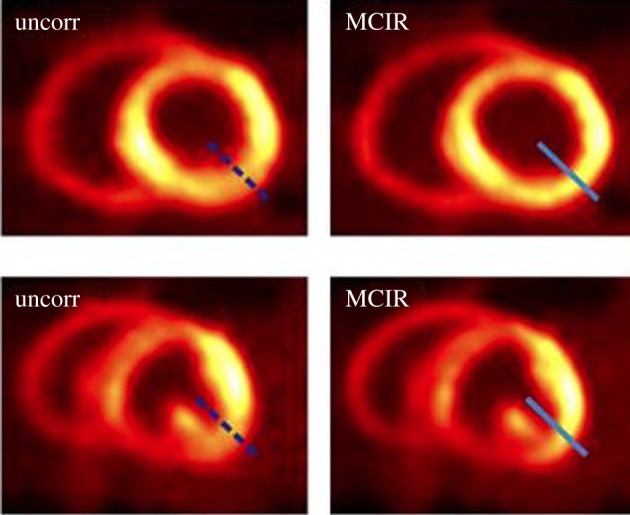
Basal (top) and midventricular (bottom) short-axis slices as shown without motion correction and with motion compensated image reconstruction. (This research was originally published in JNM. Kolbitsch *et al.* Cardiac and respiratory motion correction for simultaneous cardiac PET/MR. *The Journal of Nuclear Medicine* 2017; **58**, 846-852. © SNMMI [94]). (Online version in colour.)

Animals most commonly are anaesthetized prior to their scanning and the type of anaesthesia can affect the cardio-respiratory motion pattern. The latter can also be affected by the disease of the animal and its positioning style on the scanner. The most frequently scanned animals are mice and rats for which the diaphragm and cardiac motion cycles show a relatively similar pattern though in different spatio-temporal scales when under anaesthesia. In particular, the cardiac rate fluctuates from 300 to 600 beats per minute for mice [87] and 216 to 345 beats per minute for rats [88]. Thus, it is challenging to acquire data within such sort time frames especially for some MRI sequences. During regular mouse breathing, diaphragm motion is about 1 mm and the rib cage expansion is at 0.7 mm, consequently appropriate motion management is essential to improve PET resolution beyond the state of the art, as demonstrated by Weissler *et al.* [89]. Compensation using MRI was applied for respiratory motion on non-human primates [90] and for cardiac and respiratory motion of swine models [91–93] and around one-year-old dogs [94] showing clear improvements in image quality as illustratively demonstrated in figure 6. However, it is important to emphasize that the technical challenge to estimate motion and correct for it has not been met in small animal PET-MRI systems which are currently available in the market.

## 8. Software and test datasets

Commercial solutions for motion compensation in PET-MRI already exist. Siemens provides a solution for brain and body motion compensation for their PET-MRI systems marketed under the names BrainCOMPASS^™^ and BodyCOMPASS^™^ (Siemens Healthineers, Erlangen, Germany). With the BrainCOMPASS software, three-dimensional MR image series are acquired over time using echo planar imaging. Each acquired volume is then registered to a reference (i.e. acquired at the beginning of the scan) to estimate the motion fields. BrainCOMPASS is applied when imaging the head, while an extension of this technique named BodyCOMPASS can be applied to other regions, such as the thorax [52,95].

On the other hand, GE (General Electric Healthcare, United States) PET-MRI systems provide solutions which aim to reduce both physiological and involuntary movements with a range of motion correction approaches including PROPELLER (Periodically Rotated Overlapping Parallel Lines with Enhanced Reconstruction), PROMO (PROspective MOtion correction) and Pencil Beam Navigator. These solutions perform motion tracking sequences in MRI to track the motion during the PET scan and then sort both MRI and PET data into identical gates. Gated MRI provides the motion information used to correct PET data. PROPELLER is a navigator technique that samples strips of data in k-space and rotates them to achieve circular coverage and finally an overlapping radial sampling [96,97]. As it measures motion in the k-space, any magnetic field in-homogeneity can affect the linear trajectories during rotation even in the absence of motion. PROMO is a navigator technique [98] that uses orthogonal images in axial, transverse and coronal planes in conjunction with Kalman filter. Based on the acquired navigator signal the pulse sequence is adjusted in real time to ensure the acquisition of the field of view. PROMO is image based and therefore image accuracy affects motion tracking.

For scientists in universities, research centres and industries that develop their own prototype PET-MRI scanners, open source software packages are available that can be used to correct for motion and perform many other aspects related to image reconstruction. Some of these software solutions can also be compatible with commercial PET-MRI scanners [99,100]. More information is provided by the collaborative computational project in synergistic image reconstruction for biomedical imaging (CCP SyneRBI: https://www.ccpsynerbi.ac.uk/), which includes open source datasets (e.g. among others: https://www.isd.kcl.ac.uk/pet-mri/simulated-data/, [101]) and software for PET and MRI image reconstruction, and motion estimation and compensation [100,102,103]. What the scientific community lacks is the availability of reliable physical test objects with realistic motion capabilities mimicking human tissue deformations for all different types of motion that can be controllable externally and can be scanned with MRI, PET and CT producing realistic acquisition data and motion artefacts. Only limited custom made phantoms [104,105] have been built at this point and this is an area that lags behind the current scientific community advances and needs.

## 9. Summary and future perspective

This article offers an overview of past investigations aimed at addressing the challenging issue of management and minimization of motion-related artefacts during PET-MRI scanning. Such integrated imaging systems offer an opportunity to combine information from both modalities to increase consistency and provide mathematical constraints upon which motion can be more accurately calculated than if either machines were standalone [106]. Although external additional devices such as ECG and optical cameras are less preferable, they may be necessary in order to further increase the accuracy and validity of motion information. Information derived from PET data alone can be used for PET motion correction [107,108] and in some cases this information could be provided to motion correct MRI data as well. In cases where acceleration of an MRI acquisition is needed, while motion correction is required, deriving motion information from PET data may prove a practical and useful approach.

There has been considerable progress in motion compensation and especially in PET-MRI, and some not commonly used commercial solutions which are available on the contemporary PET-MR systems. The fact that different types of phenomena (e.g. motion, attenuation, noise in the data and other) are intertwined, the availability of a large range of radiotracers with different properties, a variety of scanning protocols (e.g. static versus dynamic), and the organs/diseases of interest in humans and animals of variable size, physiology and anatomy, makes it particularly challenging to create a universal solution [109]. A lot of hope has been placed in the use of artificial intelligence and there are some promising emerging investigations along these lines which may be able to offer solutions where the current methods, so far, have failed [110,111].

## Data accessibility

This article has no additional data.

## Disclaimer

All corresponding figures presented in the article have received appropriate copyright clearance according to the licence of their original publications.
